# Peripartum Anesthetic Management of a Patient With Sickle Cell Disease and Preeclampsia

**DOI:** 10.1002/ccr3.73662

**Published:** 2026-10-02

**Authors:** Madeline Dow, Aariya Srinivasan, Sabry Ayad

**Affiliations:** ^1^ Department of Anesthesiology Research Cleveland Clinic Foundation Cleveland Ohio USA; ^2^ Department of Anesthesiology Outcomes Research Consortium Cleveland Ohio USA; ^3^ Cleveland Clinic Fairview Hospital Cleveland Ohio USA

**Keywords:** anesthesia, hematology, obstetrics/gynecology, perinatal medicine

## Abstract

Peripartum care for patients with sickle cell disease and preeclampsia requires coordinated multidisciplinary management. Key strategies include multimodal analgesia, individualized transfusion protocols, and vigilant maternal‐fetal monitoring. Early planning and collaboration among obstetrics, hematology, anesthesiology, and palliative care are crucial for optimizing outcomes and ensuring patient safety.

## 1 Introduction

Sickle cell disease (SCD) occurs when hemoglobin S causes red blood cells to deform under hypoxic conditions and become rigid. Sickled cells obstruct the microvasculature and lead to ischemic pain, known as vaso‐occlusive crisis (VOC) [1]. In pregnancy, anemia and episodic pain are among the most common maternal complications, affecting more than 50% of pregnant individuals with SCD [1]. Physiologic changes in pregnancy, including plasma volume expansion, hypercoagulability, and increased inflammatory mediators, may heighten the risk of VOC and related complications such as acute chest syndrome and venous thrombosis in patients with SCD [2]. Preterm delivery has been reported in 9%–45% of pregnancies in patients with SCD, while fetal growth restriction occurs in approximately 8%–21% and may increase the likelihood of induced preterm birth [3].

Individuals with SCD also face an elevated risk of preeclampsia. According to the American College of Obstetricians and Gynecologists (ACOG) criteria, preeclampsia is diagnosed as new‐onset hypertension after 20 weeks of gestation with proteinuria or, in the absence of proteinuria, severe features. Severe features include systolic blood pressure ≥ 160 mmHg or diastolic blood pressure ≥ 110 mmHg, thrombocytopenia, impaired liver function, renal insufficiency, pulmonary edema, or new‐onset cerebral or visual symptoms [4]. In a large U.S. cross‐sectional study, preeclampsia occurred in 10.9% of deliveries among patients with SCD, compared with 4.5% in the study's control group [5]. For management of both preeclampsia and VOC, prevention and surveillance are critical to care. Analgesic management in patients with SCD must balance maternal pain control with fetal safety. Acetaminophen is commonly used as a first‐line therapy for VOC, whereas nonsteroidal anti‐inflammatory drugs (NSAIDs) are generally avoided later in gestation due to risks of oligohydramnios and premature ductus arteriosus closure [2, 6]. Opioids may be required for severe pain when additional analgesia is needed [7]. Prophylactic transfusion of red blood cells decreases painful crises but has not clearly improved perinatal outcomes. Because evidence guiding treatment of VOC in pregnancy remains limited, care relies on individualized multidisciplinary management [8, 9]. This case illustrates the complex peripartum anesthetic management of a primigravida with SCD complicated by VOC and preeclampsia with severe features.

## 2 Case Presentation and Examination

A 24‐year‐old primigravida patient at 33 weeks 2 days gestation with known sickle cell disease (SCD) was admitted for severe pain due to vaso‐occlusive crisis. Her additional past medical history included type 2 diabetes mellitus (T2DM), obesity, and avascular necrosis of the right knee. Her outpatient regimen consisted of buprenorphine 4, 4, and 6 mg every 8 h, and acetaminophen 1000 mg every 8 h. During the admission, palliative care was consulted for refractory pain and initiated oral morphine sulfate 10–15 mg every 4 h as needed, which was added to her regimen at discharge.

At 33 weeks 4 days gestation, she presented again with severe left thigh pain, rated 8/10 despite her ongoing medication. She reported that she had not taken her midday dose of buprenorphine. Her vitals were as follows: blood pressure 143/90 mmHg, heart rate 94 beats per minute, respiratory rate 18 breaths per minute, and oxygen saturation 97% on room air. A nonstress test was reactive, with moderate variability, accelerations present, and no decelerations. Her obstetric history was otherwise unremarkable; she denied contractions, vaginal bleeding, or membrane rupture, and fetal movement remained reassuring. She was administered 1 mg of intravenous hydromorphone with minimal improvement in pain, prompting a second 1 mg dose. Oral hydromorphone 4 mg as needed was ordered for breakthrough pain, and the patient was admitted for inpatient pain control.

## 3 Methods

Laboratory evaluation revealed low hemoglobin of 9.6 g/dL, low hematocrit of 26.2%, and elevated platelet count of 459 × 10^3^/μL. A bilateral lower‐extremity venous duplex study was negative for deep vein thrombosis. The patient was unable to tolerate MRI for further evaluation. Initial management focused on refractory vaso‐occlusive pain and anemia in anticipation of delivery. Palliative medicine followed the patient for pain management, which included patient‐controlled analgesia with intravenous hydromorphone 1 mg every 2 h as needed. As pain improved, she was transitioned from patient‐controlled analgesia to oral hydromorphone as needed for breakthrough pain while continuing buprenorphine.

The patient's anemia required early hematology involvement because a positive antibody screen and complex antibody profile made compatible packed red blood cells difficult to obtain. She received 4 units of packed red blood cells during admission to optimize hemoglobin before delivery and prepare for anticipated delivery‐related blood loss. Because compatible blood was limited, transfusion was coordinated while the patient was clinically stable rather than deferred until urgent intrapartum transfusion was needed. Intraoperative cell salvage was also planned for delivery but ultimately was not used.

During the admission, she developed preeclampsia with severe features based on repeated severe‐range systolic blood pressures, with documented blood pressures ranging from 165/86 to 167/90 mmHg. No assessment of proteinuria was documented. Laboratory data did not demonstrate renal end‐organ dysfunction, with serum creatinine 0.59 mg/dL and blood urea nitrogen 10 mg/dL. Oral nifedipine 60 mg every 12 h was initiated for blood‐pressure control, and magnesium sulfate was initiated intrapartum for seizure prophylaxis.

Given the increased risk for fetal growth abnormalities associated with SCD and T2DM, serial fetal growth surveillance and antenatal testing were performed. Estimated fetal weight was 2647 g (60th percentile) and abdominal circumference was 323.4 mm (89th percentile). Fetal ultrasound demonstrated a biophysical profile of 4/8, and delivery was recommended in the setting of abnormal fetal testing and preeclampsia with severe features.

At 35 weeks 0 days, induction of labor was initiated in the setting of abnormal fetal testing with a biophysical profile of 4/8 and preeclampsia with severe features. Despite augmentation with oxytocin, labor remained protracted at cervical dilation of 4 cm after 7 h, and the patient requested to proceed with a primary cesarean section. Labor analgesia was provided with an epidural catheter, which was redosed for cesarean delivery. Immediately after delivery, the patient reported feeling intense intraoperative pressure. Following discussion with the patient, general anesthesia was induced for completion of the procedure using fentanyl 200 μg, propofol 200 mg, succinylcholine 200 mg, and ketamine 50 mg, with additional agents including midazolam 2 mg, morphine 2.5 mg, and ketorolac 15 mg. Prophylactic agents administered perioperatively included cefazolin 3 g, azithromycin 500 mg, and tranexamic acid 1 g. Uterotonic therapy included oxytocin 10 units intravenously followed by a postpartum infusion of oxytocin 10 units. Hemodynamics remained stable during induction and emergence.

## 4 Conclusion and Results

The neonate weighed 2.565 kg and had Apgar scores of 8 and 9 at 1 and 5 min, respectively. Magnesium sulfate was continued for 24 h postpartum for seizure prophylaxis, and nifedipine 60 mg every 12 h was continued for blood pressure control. The patient reported headaches and an episode of tachycardia and shortness of breath, which improved with hydroxyzine. She was started on enoxaparin 60 mg daily for 6 weeks postpartum for venous thromboembolism prophylaxis. Following endocrinology consultation for management of T2DM, insulin was discontinued, and she was transitioned to metformin 500 mg daily. Hematology recommended maintaining hemoglobin above 10 g/dL with transfusion support as needed, continuing high‐dose folic acid supplementation, and outpatient follow‐up with her primary hematologist at discharge. By the time of discharge, her incision had healed appropriately, her pain was controlled on oral medications, and she was scheduled for multidisciplinary follow‐up with hematology, endocrinology, and obstetrics to optimize ongoing care.

## 5 Discussion

This case reflects the importance of coordinated care in the management of complex vaso‐occlusive crisis (VOC) during pregnancy complicated by preeclampsia with severe features. Pregnancy in individuals with SCD increases the risk of preeclampsia, preterm birth, and fetal growth restriction, with more than 50% of births occurring by cesarean section [3]. Common VOC management includes hydration, evaluation for precipitating factors and infection, supplemental oxygen when indicated, and analgesic therapy with opioids or other agents [1]. However, a systematic review found no definitive regimen for treating sickle cell pain crises in pregnancy [9]. Care therefore relies on early recognition of triggers, timely analgesia, appropriate fluid management, and close maternal‐fetal surveillance by a multidisciplinary team.

Analgesics in pregnancy require balancing maternal benefit and fetal risk. Acetaminophen is generally recommended as first‐line analgesia and can be dosed at usual therapeutic intervals for severe pain, although studies have raised concerns about possible risk of childhood asthma or neurodevelopmental disorders [1, 6]. NSAIDs should be avoided in late pregnancy because of risks such as premature closure of the fetal ductus arteriosus [1, 2, 6]. Patients maintained on buprenorphine should continue their maintenance therapy through labor and postpartum to reduce withdrawal risk [7]. Breakthrough pain in patients receiving buprenorphine may require additional full opioid agonists such as hydromorphone, with neonatal observation necessary for withdrawal if exposure is prolonged [1, 7]. Although there are minimal reported teratogenic risks for opioids, data remain limited [6].

Transfusions must be individualized and given in consultation with hematology due to limited evidence from trials [3, 8]. Red blood cell transfusion increases hemoglobin A and decreases the relative proportion of hemoglobin S, which reduces blood viscosity and therefore the risk of VOC [2]. Prophylactic transfusion has been associated with reductions in VOC, maternal mortality, overall pulmonary complications, and neonatal mortality, although consistent improvements have not been shown across all studies [8]. Identified transfusion risks include alloimmunization, transfusion‐related infections, and iron overload [8]. To reduce these hazards, careful planning is necessary with extended antigen matching [3, 8]. Hematology recommendations to maintain a higher hemoglobin and continue folate supplementation should guide peripartum transfusion thresholds and outpatient follow‐up [3].

Preeclampsia added obstetric urgency in our patient, requiring intensified surveillance and management. Risk factors for preeclampsia include maternal age younger than 25 or older than 45, nulliparity, obesity, and type 1 or 2 diabetes [10, 11]. Low‐dose aspirin early in pregnancy can reduce the risk of preeclampsia in high‐risk patients and should be part of preventive care when indicated [10]. Once preeclampsia develops, fetal testing helps guide timing of delivery [10]. Once preeclampsia with severe features develops, acute management requires prompt blood pressure control with antihypertensive agents such as labetalol, hydralazine, or nifedipine, alongside seizure prophylaxis with magnesium [10]. Delivery is often appropriate management for worsening preeclampsia, although postpartum monitoring remains necessary because preeclampsia may persist after delivery [11]. Postpartum follow‐up for persistent hypertension and initiation of thromboprophylaxis when indicated are crucial because patients with preeclampsia are at increased risk of cardiovascular disease later in life [11].

## Author Contributions

**Madeline Dow:** conceptualization, writing – original draft, writing – review and editing. **Aariya Srinivasan:** conceptualization, writing – original draft, writing – review and editing. **Sabry Ayad:** conceptualization, supervision, writing – review and editing.

## Funding

This research did not receive any specific grant from funding agencies in the public, commercial, or not‐for‐profit sectors.

## Consent

Written informed consent was acquired from the patient to publish this report in accordance with the journal's patient consent policy.

## Conflicts of Interest

The authors declare no conflicts of interest.

## Data Availability

The data supporting this report's findings are available on request from the corresponding author. The data is not publicly available due to privacy or ethical restrictions.
